# Active Theater as a Complementary Therapy for Parkinson's Disease Rehabilitation: A Pilot Study

**DOI:** 10.1100/tsw.2010.221

**Published:** 2010-11-16

**Authors:** Nicola Modugno, Sara Iaconelli, Mariagrazia Fiorlli, Francesco Lena, Imogen Kusch, Giovanni Mirabella

**Affiliations:** ^1^ IRCCS Neuromed, Pozzilli (IS), Italy; ^2^ Department of Experimental Medicine, University of l'Aquila, Italy

**Keywords:** art therapy, theater therapy, rehabilitation, Parkinson’s disease, nonmotor symptoms

## Abstract

Most medical treatments of Parkinson's disease (PD) are aimed at the reduction of motor symptoms. However, even when motor improvements are evident, patients often report a deterioration of their daily lives. Thus, to achieve a global improvement in personal well-being, not only drugs, but also complementary therapies, such as physical exercise, occupational and speech therapy, and active music therapy, have been used. We hypothesized that theater could reduce clinical disability and improve the quality of life of PD patients (primary end points) more efficiently than other complementary therapies because (1) in order to impersonate a character, patients are forced to regain the control of their bodies; and (2) while being part of a group, patients have a high degree of social interaction. The need to regain the control of their bodies and their social functioning is very likely to deeply motivate patients. To assess this hypothesis, we ran a randomized, controlled, and single-blinded study that lasted 3 years, on 20 subjects affected by a moderate form of idiopathic PD, in stable treatment with L-dopa and L-dopa agonists, and without severe sensory deficits. Ten patients were randomly assigned to an active theater program (in which patients were required to participate), while the others underwent physiotherapy (control group), the most common nonpharmacological treatment for PD rehabilitation. Patients of both groups were evaluated at the beginning of each year, using five clinical rating scales (Unified ParkinsonParkinson'ss Disease Rating Scale [UPDRS], Schwab and England Scale, ParkinsonParkinson'ss Disease Quality of Life [PDQ39] Scale, Epworth Sleepiness Scale, and Hamilton Depression Rating Scale). The theater patients showed progressive improvements and, at the end of the third year, they showed significant improvements in all clinical scales. Conversely, the control patients did not exhibit significant ameliorations with time. Thus, the present study provides the first scientific evidence that active theater, coupled with conventional medical treatments, represents a valid complementary therapeutic intervention for PD treatment.

### INTRODUCTION

Parkinson's disease (PD) is a neurodegenerative disease that causes motor disturbances (postural and walking difficulties, rigidity, bradykinesia, and tremor) and nonmotor disturbances, such as neuropsychiatric (e.g., depression, anxiety, cognitive impairments) and neurovegetative disorders (e.g., decreased control of urinary bladder, sexual dysfunctions; for review see [1]). Consequently, general health and the personal and social lives of patients can be deeply impaired.

Pharmacological treatment of PD, based on the administration of dopaminergic drugs, is symptomatic and mainly aimed at reducing motor symptoms. With this approach, optimal control of the symptoms is possible only in the initial stages of PD. With time, however, the positive effects become less evident, nonmotor symptoms progressively increase, side effects of the dopaminergic medications appear, and the management of the disease becomes more complex[2]. Often with the progression of the disease, PD patients experience trouble with communication and this may lead to social isolation as the person may be embarrassed by the disease (Scottish Intercollegiate Guidelines Network 2010, http://www.sign.ac.uk/index.html). As a consequence, patients start to suffer from a deterioration of their social functioning and their quality of life (QoL).

For these reasons, in addition to pharmacological treatments, complementary approaches have been tested. Most of patients undergo physiotherapy[3] and this intervention has been shown to be effective[4,5,6]. However, improvements do not always outlast the treatment[7,8] and do not always help to manage nonmotor symptoms, probably because physiotherapy is usually lacking in the motivational and emotional spheres.

Other complementary therapies, such as group psychotherapy[9], art therapies[10,11], and occupational therapies[12], have been shown to produce positive effects on QoL. These complementary therapies do not offer a cure for PD, but may help to ease the symptoms and make coping with the disease easier. In fact, complementary therapies address not only the physical aspect of PD pathology, they also tackle its social and psychological aspects. Despite this potentiality, there are few randomized controlled trials testing their efficacy[13]. Among those, Pacchetti et al.[11] showed for the first time that 3 months of active music therapy, where PD patients play an active part by using instruments and voices, improve both their motor abilities and emotional status. They attributed the improvements of motor performance, especially of bradykinesia, to the rhythmic aspect of music and to its affective arousal, which was thought to be also at the basis of ameliorations of emotional functions. Pacchetti et al.[11] concluded, suggesting that by promoting socialization, expression of feelings, awareness, and responsiveness, music therapy increases motivation in patients whose personalities are frequently characterized by apathy, anhedonia, and depressive symptoms. However, these changes were not stable. In fact, even though improvements in motor response occurred after each music therapy session, only the changes in bradykinesia showed a trend of improvement across the overall evaluation. Improvements in emotional functions were also evident after each session and at the end of the study. It is important to note that both motor and emotional changes disappeared 2 months after completion of the therapy.

We hypothesized that active theater, which shares some features with active music therapy (e.g., the combination of movements and stimulation of different sensory pathways, emotional involvement), might have a stronger effect than music because of the special features required by this discipline. In order to be able to impersonate a character, patients need to control their bodies and minds carefully. In other words, patients have to be aware of and control each movement they produce and, at the same time, they also have to represent their character's emotions, i.e., they need to be able to feel and reproduce his/her feelings. In addition, both during the performance and off the set, patients have to interact continuously, so they are forced to socialize. The strict coupling between motor control and ability to manage social situations makes theater an ideal playground to motivate patients deeply. In turn, this might allow a more stable improvement of clinical disability and QoL (primary end points) of PD patients. We compared the effect of theater therapy with physiotherapy, the most common among the nonpharmacological treatments for PD[3].

## MATERIALS AND METHODS

### Subjects

We selected 24 patients from the outpatients of the Parkinson's unit of IRCCS Neuromed Hospital (Italy). Criteria for including PD patients were: (1) being affected by a moderate form of idiopathic PD (Hoehn & Yahr stage 2–4), (2) being in stable treatment with L-dopa and L-dopa agonists, (3) not having severe sensory (visual or auditory) deficits, and (4) not having severe movement dysfunctions.

Throughout the entire course of the study, patients were allowed to continue taking their dopaminergic therapy, consisting of L-dopa alone or in association with dopamine agonists. Whenever necessary, the drug therapies were modified and optimized according to the patient's clinical progress. The assumption of antidepressant or hypnotic agents was not systematically controlled, but at the end of the study, they were equally distributed between the two groups.

Twelve patients were randomly assigned to a theater workshop rehabilitation program (*theater group*), while the other 12 underwent a standard rehabilitation therapy based on physiotherapy (*control group*). To assign a patient to a group, we adopted the following procedure. First, we matched patients two by two for age, sex, years from the PD diagnosis, and pharmacological treatments in the best possible way. Second, exploiting a computer routine implemented in MATLAB (The Mathworks, Inc., Natick, MA), we randomly assigned one member of the 12 pairs to one group. Importantly, patients knew that they were taking part in a study but, in order to prevent complaints, we did not tell to the people of one group what the other group was experiencing. None of the patients of the two groups refused to participate.

All subjects gave their informed consent and were free to withdraw from the study at any time. The general procedures were approved by the local Institutional Ethics Committee and were performed in accordance with the ethical standards laid down in the Declaration of Helsinki of 1964.

### Clinical Assessment

For the entire period of the experiment, 3 years, all patients were monitored twice a month. Four patients (two from the theater group and two from the control group) did not conclude the study because of logistic problems (they changed their home towns following their families). Two patients left the study during the first year and the other two after the second year. Their data are not included in the present study in order to allow the use of repeated-measures designs, but they were in line with those presented in the paper.

On average, the remaining patients of both groups took part in at least 70–80% of the rehabilitation sessions. All patients underwent a clinical evaluation at the beginning of the monitoring period (T0), after 1 year (T1), 2 years (T2), and 3 years (T3). A neurologist (NM) blinded to the study group evaluated the PD patients. We employed the following five clinical scales. (1) The Unified Parkinson's Disease Rating Scale (*UPDRS*) was used for rating patients' mood and cognition (UPDRS1), the activities of daily living (UPDRS2), motor symptoms (UPDRS3), and the complications of the therapy (UPDRS4). (2) The Parkinson's Disease Quality of Life Scale (*PDQ39*) measured the QoL by summoning the scores of its eight subscales (*mobility*, a*ctivities of daily living*, e*motional well-being*, s*tigma*, s*ocial support*, c*ognition*, c*ommunication*, and *bodily discomfort)*. (3) The *Epworth Sleepiness Scale* (measuring the level of daytime drowsiness) and (4) the *Hamilton Depression Rating Scale* (measuring the level of depression) were also used for rating nonmotor symptoms. (5) The *Schwab and England Scale* was used to assess the degree of functional independence in daily living. We employed several clinical scales because we wanted to assess the overall QoL of patients from different points of view.

The ratings of all scales were always carried out in their dopaminergic "ON" state. We assessed it by asking each patient and his relatives (1) "What is the amount of the first morning dose that normally allows him/her to reach the best control of symptoms?" and (2) "How long does it take to reach the 'ON'

state?" As a result, patients were rated 30–60 min after the administration of a dose of L-dopa ranging from 100 to 200 mg.

### Physiotherapy

Physiotherapy consisted of individual 2- to 3-h daily sessions, 3 days/week (~18 h/month), for 3 years, with two physiotherapists. Aims of this therapy were: preventing osteoarticular rigidity, muscular contractures, and atrophies; grading postural abnormalities; promoting postural reactions; promoting postural changes; training balance and gait; and increasing tolerance to the physical exercise (for more details see Table 1).

**TABLE 1**
**Exercises Included in the Physiotherapy Rehabilitation Program**

| Activities | Basic Exercises* |
|---|---|
| Cardiovascular warm up (10 min) | Intersegmental coordination exercises |
| | Exercises to release shoulder and pelvic girdle |
| | Pelvic anteversion and retroversion movements to stimulate diaphragmatic respiration (supine position) |
| | Breathing exercises to promote expansion of the chest |
| Stretching exercises (15 min) | Exercises to stretch the muscles of the posterior kinematic chain |
| | Exercises to stretch the pectoralis muscles |
| | Exercises to stretch the ischio-cruralis muscles |
| | Assumption of the prone position, sitting on heels and stretching the arms out in front |
| | Exercises to stretch lumbar muscles (in the supine position, each knee, in turn, is brought to the chest) |
| Postural exercises (15 min) | Anterior and lateral flexion, extension and rotation of the trunk in standing position, to promote spinal column mobilization |
| Overground gait training (20 min) | Overground gait training (forwards, backwards, and lateral) |
| | Walking on the spot |
| Balance training (15 min) | Path with obstacles |
| | Balance exercises performed in order of difficulty: |
| | Heel-to-toe walking |
| | Lateral walking, crossing the legs |
| | Walking along a path on surfaces of different texture (foam mats, mats containing sand, etc.) |
| Relaxation exercises (15 min) | Intersegmental coordination exercises |
| | Segmental passive mobilization (until maximum joint range of motion is reached) |
| | Breathing exercises to promote expansion of the chest |

* Depending on the single patient's motor and functional status, the physiotherapist could include exercises other than those indicated as basic exercises.

### Therapeutic Theater Workshop

The theatrical workshop, led by the theater company, consisted of 6-h daily sessions, for two consecutive days, once or twice per month, for a total of ~18 h/months, for 3 years (for more details see Table 2). The initial part of every workshop focused on exercising basic skills. All subjects were trained in controlling breathing, posture, gait, coordination, and manual tasks.

(MOVIE S1: This movie shows some exercises of motor coordination performed by one PD patient, with 1 year of theater training, together with an actress during one theatrical workshop session. The patient was in "ON" therapy.)

**TABLE 2**
**Exercises Included in a Typical Theater Training Session**

| Activities | Basic Exercises* |
|---|---|
| Vocal warm up (20 min) | Feet on the ground, erect position on the chairs |
| | Massage and tapping of the chest |
| | Hands on the belly, movement of abdominal muscles |
| | Diaphragmatic breathing |
| | Vocal emission of the consonant "S" |
| | Vocal emission of the consonant "M", keeping mouth closed |
| | Vocal emission of the syllable "NO", low note, insisting on the projection of the consonant as a springboard for the vowel |
| | Emission of the syllables PA, PE, PI, PO, PU |
| | Emission of the consonant "M", keeping mouth closed, but gradually rising note and back |
| | Emission of the syllable "MA", but gradually rising note and back |
| | Vocal emission of the breath, freely moving the facemask |
| | Rotation of the tongue inside and outside of teeth |
| | Emission of the vowels keeping the tongue out of the mouth |
| | Emission of the vowels, exasperating facial expression |
| | Emission of all the vowels while laughing |
| Preparation of the scene (40 min): to be performed in a circle, always repeating the same line to the person beside them | Slow vocal articulation of a line from the scene |
| | Projection of the line into the distance, leaning the sound on the consonant |
| | Articulation of the line in a low voice |
| | Repeating the line with a different rhythm |
| | Attempt to speak the line in five different emotional moods (anger, fear, sensuality, happiness, sadness) |
| | In circle, adding the second line to the first |
| | Attempt to speak the two lines in five different moods (see above) |
| | Continue the same exercise, adding lines of the script depending on the scene |
| Staging (5 h) | After having created an acting and an audience area, the involved patients perform the scene they were previously exercising technically |

* Some lessons could be preceded by a video (10 min) of the scene to be performed.

The patients were then taught to approach theater texts and to analyze them. In the second part of the workshop, patients rehearsed singly or in groups, together with actors, based on improvisation or sketches. Sketches were always directed by actors of the company with the aim of recreating on the stage behaviors and emotions that could occur in real life. Through this training, patients learned to express themselves and to communicate with each other both verbally and nonverbally. After periods of training, some of the patients wrote a script and eventually presented it with the help of the director.

(MOVIE S2: This movie shows a sketch where a PD patient, with 1 year of theater training, impersonates a man who finds out that he has won the lottery. The patient was in "ON" therapy.)

### Statistics

We employed the Shapiro-Wilk test of normality in order to assess whether a given sample came from a normally distributed population.

Analyses of variance (ANOVAs) were employed for assessing (1) changes in drug therapy at T0 vs. T3 in theater vs. control group and (2) changes over time (T0, T1, T2, T3) in theater vs. control groups across all clinical scales. Mauchley's test evaluated the sphericity assumption and, where appropriate, correction of the degrees of freedom was made according to the Greenhouse-Geisser procedure. Bonferroni correction was applied to all posthoc tests (pairwise comparisons).

In order to control for the sample size, for each statistic we computed the partial eta-squared ($\eta^2$), a coefficient that estimates the so-called "effect size". The effect size is a measure used for describing the degree of relationship between dependent and independent variables, independently from the sample size. Values of $\eta^2$ higher than 0.14 indicate strong effect sizes, namely that the F values obtained are unlikely to depend on the sample size. Values of $\eta^2$ smaller than 0.06 indicate small effect sizes.

### RESULTS

At time T0, i.e., prior to the theatrical experience, all clinical parameters of the two groups (see Table 3) were homogeneous. T-tests revealed no significant differences for age (t[18] = 0.618, $p$ = 0.544), years with diagnosis (t[18] = –0.047, $p$ = 0.963), or Hoehn and Yahr score (t[18] = 0.361, $p$ = 0.722). Furthermore, an ANOVA on the amount of L-dopa equivalents/kg administered, with time (T0 and T3) and group (theater and control) as factors, showed a significant main effect of time (two-way repeated-measures ANOVA F[1] = 6.564, $p < 0.05$), but no main effect of group (F[1] = 0.582, $p$ = 0.455) and of interaction (F[1] = 3.1, $p$ = 0.09). However, even though there was no interaction between time and group (it was very close to be significant), theater-group patients did not require an increase in dopaminergic drug therapy during the course of the study, while the control patients did.

**TABLE 3**
**Clinical Data of Patients Affected by PD of Control (CTRL) and Theater (TH) Group***

| Patient | Sex | | Age | | Hoehn & Yahr T0 | | Years with Diagnosis | | L-DOPA EQ. T0 | | L-DOPA EQ. T3 | |
|---|---|---|---|---|---|---|---|---|---|---|---|---|
| | CTRL | TH | CTRL | TH | CTRL | TH | CTRL | TH | CTRL | TH | CTRL | TH |
| 1 | F | F | 70 | 58 | 4 | 3 | 16 | 24 | 3650 | 905 | 3800 | 977 |
| 2 | M | M | 64 | 58 | 4 | 4 | 12 | 7 | 3400 | 1603 | 3600 | 1403 |
| 3 | F | F | 65 | 61 | 4 | 4 | 13 | 6 | 1105 | 977 | 1141 | 877 |
| 4 | F | F | 62 | 72 | 4 | 4 | 9 | 12 | 805 | 3400 | 705 | 3400 |
| 5 | M | M | 59 | 65 | 3 | 3 | 5 | 8 | 200 | 1263 | 344 | 1263 |
| 6 | M | M | 63 | 66 | 4 | 4 | 10 | 11 | 3350 | 1563 | 3600 | 1707 |
| 7 | F | F | 65 | 62 | 3 | 3 | 6 | 5 | 561 | 1263 | 661 | 1263 |
| 8 | M | M | 65 | 63 | 3 | 3 | 9 | 10 | 1105 | 1543 | 1255 | 1507 |
| 9 | M | M | 62 | 61 | 3 | 4 | 6 | 4 | 1363 | 566 | 1363 | 766 |
| 10 | F | F | 57 | 54 | 3 | 2 | 8 | 8 | 1263 | 441 | 1407 | 561 |

| | | | | | | | | | | |
|---|---|---|---|---|---|---|---|---|---|---|
| Mean (SEM) | 63.2 (1.13) | 62 (1.58) | 3.5 (0.17) | 3 (0.22) | 9.4 (1.1) | 10 (1.8) | 1680.3 (405.1) | 1352.4 (261.2) | 1787.6 (423.8) | 1372 (251.5) |
| t-test | *p* = 0.544 | | *p* = 0.722 | | *p* = 0.963 | | See Results (ANOVA) | | | |

* For each patient, sex, age, Hoehn and Yahr scores (indicating the relative level of disability due to PD disease) at time point T0 in the phase "OFF" (after the suspension of anti-Parkinson therapy for 12 h), years with diagnosis, L-dopa equivalents/kg at time T0, and L-dopa equivalents/kg at time T3 are given. At the bottom of the table, the *p* values for the comparisons between the two groups are reported.

In order to compare the scores obtained in the clinical scales by the two groups, first we checked whether data were normally distributed. Results are reported in Table 4. As can be noted in the large majority of instances (107/120 or 89.2%), the Shapiro-Wilk test was not significant, indicating that the assumption of normality could not be refused. Thus, we decided to apply parametric rather than nonparametric statistics because the former are much more robust.

**TABLE 4**
**Results of Shapiro-Wilk Test of Normality Obtained for the Two Groups of PD Patients (Control Group and Theater Group) in all Clinical Scales (and Subscales) at the Four Time Points***

| | **Control (*p* Values)** | | | | **Theater (*p* Values)** | | | |
|---|---|---|---|---|---|---|---|---|
| | **T0** | **T1** | **T2** | **T3** | **T0** | **T1** | **T2** | **T3** |
| Epworth Sleepiness | = 0.481 | = 0.331 | = 0.263 | = 0.517 | = 0.643 | = 0.305 | = 0.680 | = 0.892 |
| Hamilton | = 0.692 | = 0.251 | = 0.198 | = 0.101 | = 0.078 | = 0.837 | = 0.630 | **< 0.01** |
| Schwab and England | = 0.287 | = 0.191 | = 0.389 | **< 0.05** | = 0.321 | **< 0.05** | = 0.124 | **< 0.05** |
| UPDRS1 | = 0.247 | = 0.295 | = 0.636 | = 0.576 | = 0.140 | = 0.496 | = 0.392 | = 0.258 |
| UPDRS2 | = 0.299 | = 0.481 | = 0.554 | = 0.887 | = 0.618 | = 0.728 | = 0.769 | = 0.882 |
| UPDRS3 | = 0.737 | = 0.759 | = 0.574 | = 0.505 | = 0.161 | **< 0.05** | = 0.099 | **< 0.05** |
| UPDRS4 | = 0.683 | = 0.252 | = 0.073 | = 0.084 | = 0.321 | = 0.417 | = 0.750 | = 0.569 |
| Mobility** | = 0.144 | = 0.126 | = 0.307 | = 0.257 | = 0.866 | = 0.252 | = 0.999 | = 0.197 |
| Activities of daily living** | = 0.883 | = 0.601 | = 0.101 | = 0.238 | = 0.071 | = 0.183 | = 0.241 | = 0.458 |
| Emotional well-being** | = 0.137 | = 0.198 | = 0.519 | **< 0.01** | = 0.447 | = 0.259 | = 0.415 | = 0.092 |
| Stigma** | = 0.186 | = 0.528 | = 0.457 | **< 0.05** | = 0.767 | = 0.321 | = 0.344 | = 0.550 |
| Social support** | = 0.245 | = 0.158 | = 0.191 | = 0.272 | **< 0.05** | = 0.717 | **< 0.05** | = 0.108 |
| Cognition** | = 0.627 | = 0.073 | = 0.516 | = 0.113 | = 0.106 | = 0.555 | = 0.072 | **< 0.05** |
| Communication** | = 0.295 | = 0.198 | = 0.138 | = 0.532 | = 0.053 | = 0.140 | = 0.156 | **< 0.05** |
| Bodily discomfort** | = 0.099 | = 0.092 | = 0.344 | **< 0.01** | = 0.241 | = 0.272 | = 0.062 | = 0.191 |

* The Shapiro-Wilk test tests the null hypothesis that a given sample came from a normally distributed population. Thus, *p* values less than 0.05 indicate that the data are not from a normally distributed population and are indicated in bold.

** PDQ39 subscales.

The effect of theatrical training is illustrated in Tables 5 and 6, Figs. 1 and 2. To assess differences for the score of each clinical scale, we employed a doubly multivariate repeated-measures design with time (T0, T1, T2, T3) and group (control, theater) as factors. Results are summarized in Table 6.

(MOVIE S3: This movie shows a PD patient performing a singing exercise at time T0 [at the beginning of theatrical workshop] and at time T1 [after 1 year of theater training]. Clearly, at T0, the patient is impaired, while at T1, he is able to perform the exercise without effort. In both instances, the patient was in "ON" therapy.)

(MOVIE S4: This movie shows two sketches played by a PD patient at T1 [after 1 year of theatrical training] and at T3 [after 3 years of theatrical training]. In the first sketch, the patient [who is in front of the camera, wearing a black T-shirt] is acting the part of a man who is altercating with two other people [another PD patient and an actress]. In the second sketch, the same patient [wearing a black shirt] performs some karate moves, leading the group choreography. It is worth noticing: [1] the much greater mobility and body control; [2] the increased expressivity; [3] the remarkable weight loss of the patient in the second sketch with respect to the first one. In both instances, the patient was in "ON" therapy.)

**TABLE 5**
**Mean Scores and Standard Error Mean Obtained by the Two Groups of PD Patients (Control Group and Theater Group) in the Five Clinical Scales (and Subscales) at the Four Time Points**

| | Control | | | | Theater | | | |
|---|---|---|---|---|---|---|---|---|
| | T0 | T1 | T2 | T3 | T0 | T1 | T2 | T3 |
| Epworth Sleepiness | 9.9 ± 0.74 | 9.2 ± 0.87 | 9 ± 1.17 | 9.6 ± 1.08 | 11 ± 0.83 | 7 ± 0.97 | 6 ± 1.13 | 4 ± 0.86 |
| Hamilton | 16.8 ± 1.96 | 18.7 ± 2.49 | 18.3 ± 2.44 | 17.7 ± 2.50 | 24 ± 1.23 | 19.6 ± 1.53 | 16.1 ± 1.55 | 11.7 ± 1.12 |
| Schwab and England | 67 ± 3.00 | 72 ± 3.27 | 72 ± 3.89 | 75 ± 5.22 | 62 ± 4.90 | 66 ± 4.00 | 74 ± 3.71 | 79 ± 3.79 |
| UPDRS1 | 4.1 ± 0.84 | 4.3 ± 0.88 | 4.4 ± 0.91 | 4.3 ± 0.91 | 2.3 ± 0.50 | 2.5 ± 0.62 | 1.9 ± 0.41 | 1.5 ± 0.34 |
| UPDRS2 | 16.6 ± 2.03 | 15.5 ± 1.93 | 15.7 ± 2.02 | 15.6 ± 2.10 | 14.6 ± 2.58 | 14.6 ± 2.67 | 13.7 ± 2.33 | 13.3 ± 2.31 |
| UPDRS3 | 23.5 ± 3.01 | 22.1 ± 3.04 | 22.6 ± 3.29 | 21.7 ± 3.33 | 26.9 ± 4.86 | 23.4 ± 5.29 | 21.2 ± 4.30 | 19.5 ± 4.03 |
| UPDRS4 | 5.9 ± 0.95 | 6.2 ± 1.24 | 6.8 ± 1.37 | 7.2 ± 1.54 | 7.4 ± 0.69 | 7.2 ± 1.02 | 6.7 ± 1.01 | 6.5 ± 1.12 |
| Mobility* | 11.1 ± 1.43 | 10.6 ± 1.25 | 9.4 ± 1.23 | 9.3 ± 1.45 | 11.7 ± 1.58 | 11.6 ± 1.43 | 9.2 ± 1.28 | 7.6 ± 1.07 |
| Activities of daily living* | 7.1 ± 0.60 | 6.3 ± 0.72 | 6.0 ± 0.77 | 5.7 ± 1.01 | 8.4 ± 1.49 | 7.9 ± 1.20 | 6.6 ± 0.45 | 4.7 ± 0.68 |
| Emotional well-being* | 6.6 ± 0.78 | 5.8 ± 0.93 | 5.7 ± 0.86 | 5.2 ± 0.80 | 7.7 ± 0.99 | 7.0 ± 0.77 | 5.8 ± 0.90 | 3.9 ± 0.85 |
| Stigma* | 3.3 ± 0.56 | 3.1 ± 0.60 | 3.1 ± 0.53 | 3.2 ± 0.66 | 3.1 ± 0.67 | 3.2 ± 0.49 | 2.0 ± 0.42 | 2.0 ± 0.39 |
| Social support* | 3.6 ± 0.31 | 3.0 ± 0.45 | 2.8 ± 0.33 | 2.5 ± 0.54 | 6.2 ± 0.66 | 4.6 ± 0.64 | 3.6 ± 0.52 | 2.4 ± 0.37 |
| Cognition* | 4.7 ± 0.52 | 4.0 ± 0.52 | 3.7 ± 0.58 | 3.6 ± 0.69 | 5.7 ± 0.56 | 5.4 ± 0.62 | 4.7 ± 0.63 | 3.6 ± 0.43 |
| Communication* | 3.1 ± 0.50 | 2.4 ± 0.43 | 2.7 ± 0.47 | 2.4 ± 0.52 | 4.2 ± 0.63 | 4.3 ± 0.50 | 3.8 ± 0.49 | 2.9 ± 0.23 |
| Bodily discomfort* | 4.2 ± 0.55 | 3.1 ± 0.46 | 3.0 ± 0.42 | 2.9 ± 0.48 | 5.3 ± 0.68 | 5.0 ± 0.71 | 3.6 ± 0.48 | 2.8 ± 0.33 |

* PDQ39 subscales.

**TABLE 6**
**Results of the Doubly Multivariate Repeated-Measures ANOVA with Group (Control, Theater) and Time (T0, T1, T2, T3) as Factors in the Clinical Scales (or Subscales)**

| | Time | | | Group | | | Time × Group | | |
|---|---|---|---|---|---|---|---|---|---|
| | F (df) | Sig.* | $\eta^2$ | F (df) | Sig.* | $\eta^2$ | F (df) | Sig.* | $\eta^2$ |
| Epworth Sleepiness | F(1.5,28.1) = 27.28 | **< 0.001** | 0.603 | F(1,18) = 3.47 | = 0.07 | 0.162 | F(1.5,28.1) = 19.99 | **< 0.001** | 0.526 |
| Hamilton | F(3,54) = 40.3 | **< 0.001** | 0.691 | F(1,18) = 0.00 | = 0.99 | 0.000 | F(3,54) = 50.81 | **< 0.001** | 0.738 |
| Schwab and England | F(2.1,38.7) = 21 | **< 0.001** | 0.538 | F(1,18) = 0.55 | = 0.81 | 0.003 | F(2.1,38.7) = 4.55 | **< 0.05** | 0.202 |
| UPDRS1 | F(1.9,35.9) = 2.62 | = 0.09 | 0.127 | F(1,18) = 5.15 | **< 0.05** | 0.222 | F(1.9,35.9) = 3.97 | **< 0.05** | 0.181 |
| UPDRS2 | F(2.1,37.1) = 3.35 | **< 0.05** | 0.157 | F(1,18) = 0.32 | = 0.57 | 0.018 | F(2.1,37.1) = 1.28 | = 0.29 | 0.067 |
| UPDRS3 | F(1.8,33.3) = 8.24 | **< 0.01** | 0.314 | F(1,18) = 0.00 | = 0.95 | 0.000 | F(1.8,33.3) = 3.62 | **< 0.05** | 0.168 |
| UPDRS4 | F(1.4,25.2) = 0.11 | = 0.82 | 0.006 | F(1,18) = 0.07 | = 0.78 | 0.004 | F(1.4,25.2) = 3.88 | **< 0.05** | 0.177 |
| Mobility** | F(1.3,24.6) = 11.77 | **< 0.001** | 0.395 | F(1,18) = 0.02 | = 0.96 | 0.000 | F(1.3,24.6) = 2.07 | = 0.15 | 0.103 |
| Activities of daily living** | F(1.3,23.9) = 8.46 | **< 0.01** | 0.320 | F(1,18) = 0.3 | = 0.58 | 0.017 | F(1.3,23.9) = 2.36 | = 0.13 | 0.116 |
| Emotional well-being** | F(1.9,35.7) = 15.48 | **< 0.001** | 0.462 | F(1,18) = 0.6 | = 0.81 | 0.003 | F(1.9,35.7) = 4.3 | **< 0.05** | 0.193 |
| Stigma** | F(3,54) = 3.18 | **< 0.05** | 0.150 | F(1,18) = 0.73 | = 0.40 | 0.039 | F(3,54) = 2.76 | **< 0.05** | 0.133 |

| | | | | | | | | | |
|---|---|---|---|---|---|---|---|---|---|
| Social support** | F(3,54) = 22.59 | **< 0.001** | 0.557 | F(1,18) = 4.35 | = 0.051 | 0.195 | F(3,54) = 6.97 | **< 0.001** | 0.279 |
| Cognition** | F(1.9,35.7) = 9.93 | **< 0.001** | 0.356 | F(1,18) = 1.40 | = 0.25 | 0.073 | F(1.9,35.7) = 1.88 | = 0.16 | 0.095 |
| Communication** | F(1.8,33.3) = 4.46 | **< 0.05** | 0.199 | F(1,18) = 3.79 | = 0.07 | 0.174 | F(1.8,33.3) = 2.09 | = 0.14 | 0.104 |
| Bodily discomfort** | F(1.6,29.6) = 13.73 | **< 0.001** | 0.433 | F(1,18) = 1.88 | = 0.19 | 0.095 | F(1.6,29.6) = 3.44 | = 0.052 | 0.162 |

* *p* values.

** PDQ39 subscales.

$\eta^2$ represents the partial eta-squared, values higher than 0.14 indicate strong effect sizes; namely, that the F values obtained are unlikely to depend on the sample size (see Materials and Methods).

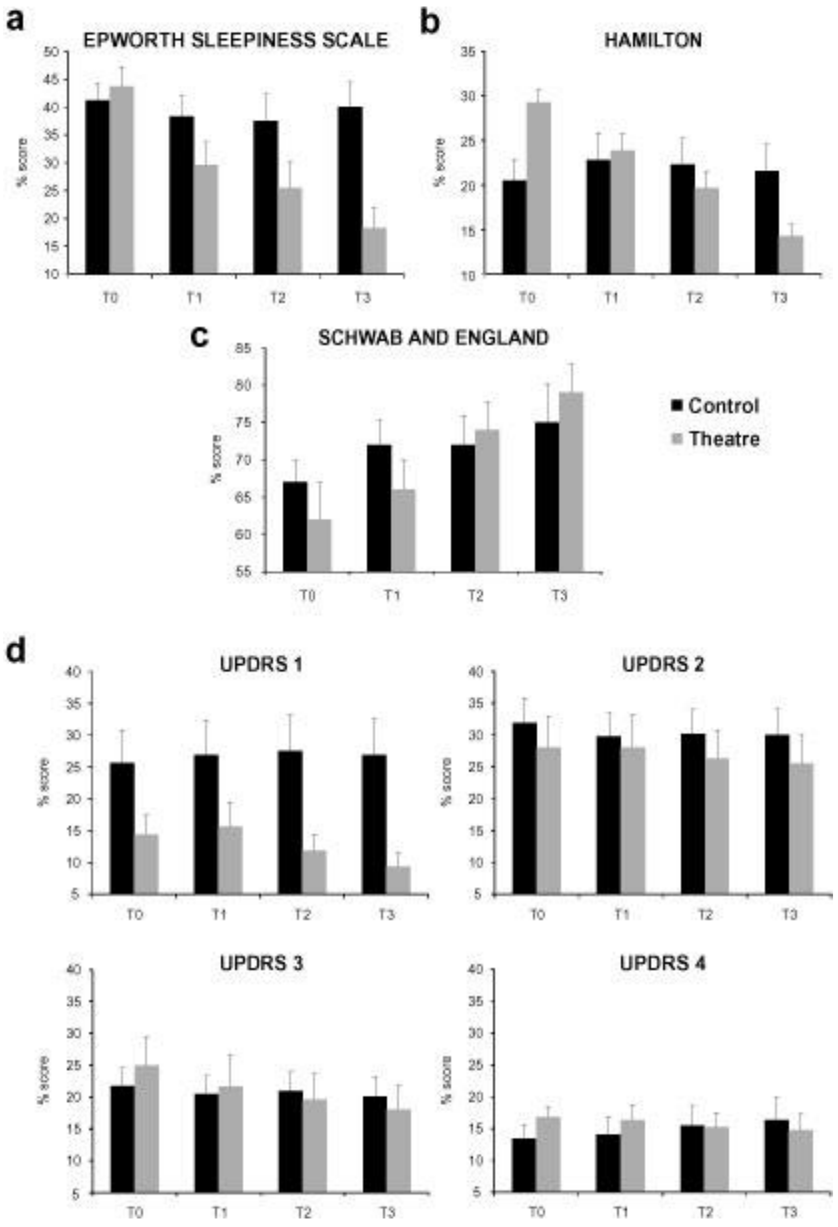

**FIGURE 1.** Mean scores and standard error of the means at the four time points in the Epworth Sleepiness Scale (A), Hamilton Depression Rating Scale (B), Schwab and England Scale (C), and in the four subscales of the UPDRS (D). All values are reported as percentage with respect to the maximum of the scale. Patients were tested in the "ON" state (see Material and Methods for more details).

We found a significant main effect of the factor time in all clinical scales except for UPDRS1 and UPDRS4. This indicates that in the majority of the scales, a significant change occurred at some point during the 3 years of the study. Posthoc pairwise comparisons showed that the mean scores of nonmotor clinical scales tended to change early on; namely, often they were already different after the first year (T0 vs. T1), while the improvement was less fast for UPDRS3, in which the first significant difference could be observed after the second year.

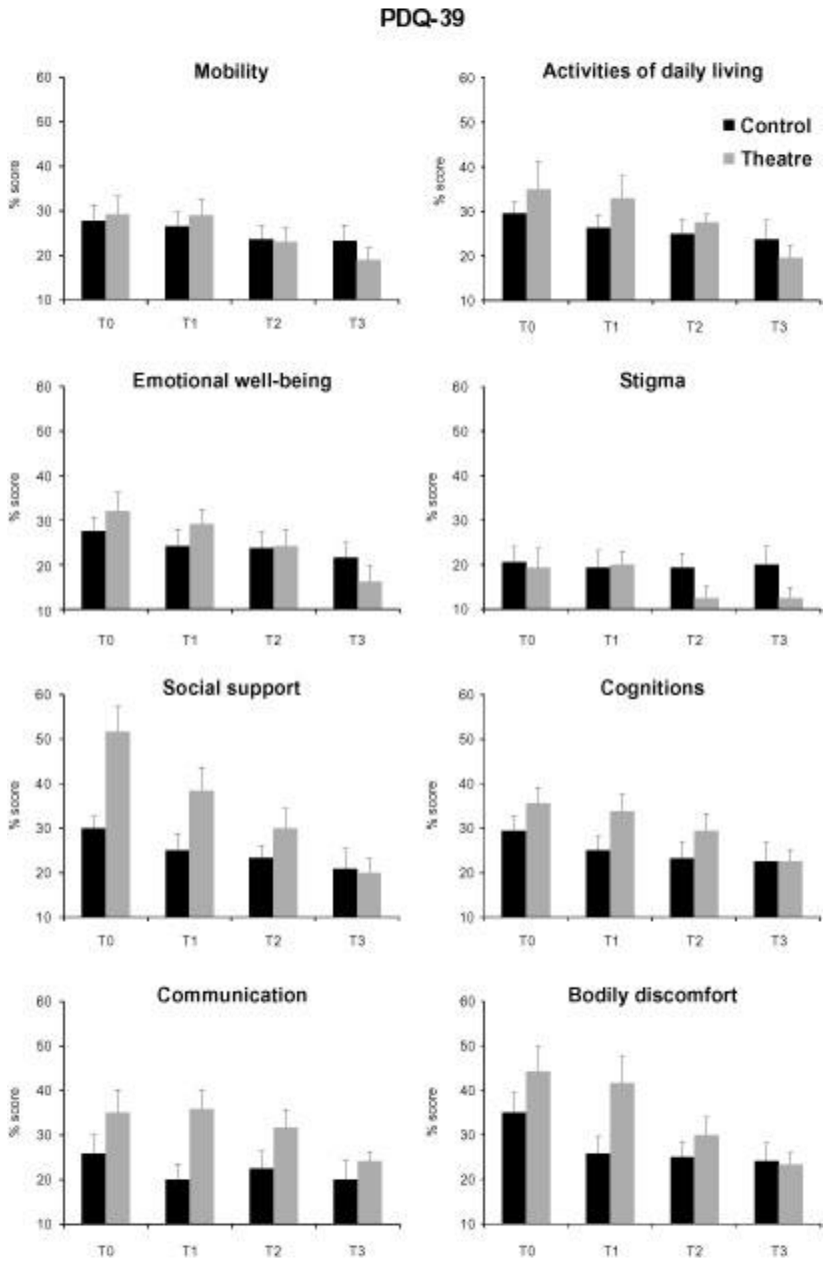

**FIGURE 2**. Mean scores and standard error of the means at the four time points for the theater (gray bars) and control group (black bars) in the eight subscales of the PDQ39. All values are reported as percentage with respect to the maximum of the scale. Patients were tested in the "ON" state (see Material and Methods for more details).

The factor group was significant only for the UPDRS1 scale. Posthoc pairwise comparisons on the factor group revealed that the mean differences between scores of the control and theater groups became significantly different at T2 and T3 only. This means that at T0 and T1, there was not a significant difference in patients' mood and cognition between the two groups.

Finally a significant interaction was found in the Epworth Sleepiness Scale, Hamilton Depression Rating Scales, Schwab and England Scale, UPDRS1, UPDRS3, and UPDRS4 scales, and for three subscales of PDQ39 (emotional well-being, stigma, and social support). Posthoc pairwise comparisons revealed that (1) PD theater patients showed a significant improvement in most of the nonmotor clinical scales and, to a lesser extent, in the UPDRS3; on the other hand, the scores of the control group remained substantially unchanged; (2) in the theater group, the improvement in nonmotor clinical scales took place faster than those in the motor scale, which became evident only after 2 years.

Remarkably, with one only exception, the $\eta^2$ was always higher than 0.14 when the F values were significant (see Table 6). This finding underlines that in all those instances, there were strong effect sizes; namely, that the F values obtained did not depend on the small sample size.

To summarize, at T0, the cognitive status of the two groups was homogeneous (at T0 the UPDRS1, the PDQ39 emotional well-being, and the PDQ39 stigma were not significantly different). The only significant difference was related to the feeling of social support; patients of the theater group felt less supported than controls. Nonmotor symptoms of PD patients of the theater group improved over time more consistently than motor symptoms. Of relevance, they showed a very quick improvement in the mood, quality of sleep, and perception of social support (as revealed by the significant improvement of the Hamilton Depression Rating Scale, Epworth Sleepiness Scale, and PDQ39 social support scale scores at T1 with respect to T0). All these parameters further improved at T2. In addition, significant ameliorations of functional independence, emotional well-being, and stigma were assessed at the same time. Finally, at T2, motor symptoms also significantly improved. Thus, our results show that the theater group had a clear improvement over time, while the control group did not display significant improvements during the 3 years with respect to T0.

### DISCUSSION

The idea of PD as a pure motor disturbance must be reconsidered. Phenomena like kinesia paradoxica, i.e., the sudden mobility and agility of otherwise akinetic PD patients in situations of great emergency, suggest that the nature of this pathology cannot be motor in a strict sense. In line with this hypothesis, Mazzoni et al.[14] showed that patients can exhibit "normal" motor behavior even in simple arm-reaching movements, not just in extreme cases. Clinical observations of PD progression also reveal the negative effects of nonmotor symptoms on patient well-being[15]. Often a patient's personality traits change: calm and quiet people, after developing PD, can start doing things they never did before, such as writing or painting compulsively[16,17]. Patients might even lose the ability to estimate the consequences of their actions correctly, developing compulsive reward-seeking activities (e.g., gambling[18]). About 50% of PD patients suffer from apathy or anhedonia[19], which leads to loss of motivation and ability to plan daily life.

Despite this evidence, nonmotor features are usually under-recognized and undertreated because attention is paid to motor symptoms that are treated with dopaminergic drugs. This approach can lead to the paradoxical discrepancy between an objectively good control of motor disturbances and an increasingly negative feeling of well-being reported by the patient. Thus, to achieve global improvement in personal well-being, complementary approaches are needed, but only a few have been explored.

Even though physiotherapy has been shown to be effective in improving patients' physical performance and QoL[20], benefits are often temporary[7,8]. Occupational therapies, art therapies (such as music[11] and dance[10]), and group psychotherapy[9] have also been shown to produce positive effects on QoL, anxiety, and depression. However, at the moment, it is not possible to formulate recommendations for those approaches because there is too few evidence of positive effects. For instance, Pacchetti et al.[11] showed that active music therapy improves both motor abilities and the emotional status of PD patients; however, improvements tended to disappear within a few months after completion of the therapy.

On the other hand, our study shows for the first time that active theater has positive and stable effects on the cognitive, affective, and motor domains of PD patients, thus improving their overall QoL. The greater impact of our theater training with respect to previously employed complementary therapies could be explained in two ways that are likely to interact with each other. First, our rehabilitation program had a longer duration than previous programs (i.e., the active music therapy of Pacchetti et al.[11] lasted only 3 months). Second, theater requires the development of peculiar abilities. To perform on stage, patients are forced to control their movements, thoughts, and emotions carefully. Furthermore, theater requires a high degree of personal interaction and thus promotes socialization, lessening the feeling of isolation frequently reported by PD patients. Possibly, thanks to all these elements, active theater deeply motivates patients, allowing them to regain self-confidence and to develop higher self-control.

### LIMITATIONS OF THE STUDY

We are aware of methodological limitations of our study and, on this basis, we believe that it has to be considered a pilot study. First of all, the sample is relatively small (but the low number of subjects was needed in order to allow a rigorous intervention on each subject for a long period of time and we controlled that our results did not depend on the small sample size). Second, as stated in the Materials and Methods section, we carried out this experiment only on patients affected by a moderate form of PD (Hoehn & Yahr stage 2–4). The rationale was that since mild PD patients have less motor symptoms, they could fully live the theatrical experience and we thus thought it would be simpler to observe differences with respect to the control group. Currently, we do not know whether the most severe cases of PD could benefit by the theatrical experience. This will be the topic of future researches. Third, we are aware that our theater therapy program might not be easily transferred to different hospitals or clinics.

Nevertheless, this first quantitative demonstration of the efficacy of active theater as a rehabilitative tool for PD could open the doors to other clinical trials and confirm that the use of complementary approaches can bridge the gap between the best effect of the pharmacological therapy and the achievement of a better QoL.

### ACKNOWLEDGMENTS

We would like to acknowledge the contribution of the theater therapy company Klesidra for people with neurodegenerative diseases (http://www.klesidra.org). We are grateful to M. Manfredi, S. Ruggeri, and G. Cantore for support throughout this research.

This research was supported by the Italian Ministry of Work, Health and Social Policies (Bando Giovani Ricercatori 2007) and by the Italian Ministry of University and Research (PRIN n.2008_RBFNLH_005).

## References

1. Chaudhuri, K.R. and Schapira, A.H. (2009) Non-motor symptoms of Parkinson's disease: dopaminergic pathophysiology and treatment. *Lancet Neurol*. **8,** 464–474.
2. Yarrow, S. (1999) Members' 1998 survey of the Parkinson Disease Society of the United Kingdom. In *Parkinson's Disease: Studies in Psychological and Social Care*. Percival, R. and Hobson, P., Eds. BPS Bookx, Leicester. pp. 79–92.
3. Bohannon, R.W. (1993) Physical rehabilitation in neurologic diseases. *Curr. Opin. Neurol*. **6,** 765–772.
4. Guo, L., Jiang, Y., Yatsuya, H., Yoshida, Y., and Sakamoto, J. (2009) Group education with personal rehabilitation for idiopathic Parkinson's disease. *Can. J. Neurol. Sci.* **36,** 51–59.
5. Tassorelli, C., Buscone, S., Sandrini, G., Pacchetti, C., Furnari, A., Zangaglia, R., Bartolo, M., Nappi, G., and Martignoni, E. (2009) The role of rehabilitation in deep brain stimulation of the subthalamic nucleus for Parkinson's disease: a pilot study. *Parkinsonism Relat. Disord.* **15(9),** 675–681.
6. Tickle-Degnen, L., Ellis, T., Saint-Hilaire, M.H., Thomas, C.A., and Wagenaar, R.C. (2010) Self-management rehabilitation and health-related quality of life in Parkinson's disease: a randomized controlled trial. *Mov. Disord*. **25,** 194–204.
7. Carne, W., Cifu, D.X., Marcinko, P., Baron, M., Pickett, T., Qutubuddin, A., Calabrese, V., Roberge, P., Holloway, K., and Mutchler, B. (2005) Efficacy of multidisciplinary treatment program on long-term outcomes of individuals with Parkinson's disease. *J. Rehabil. Res. Dev.* **42,** 779–786.
8. Reuter, I., Engelhardt, M., Stecker, K., and Baas, H. (1999) Therapeutic value of exercise training in Parkinson's disease. *Med. Sci. Sports Exerc*. **31,** 1544–1549.
9. Sproesser, E., Viana, M.A., Quagliato, E.M., and de Souza, E.A. (2010) The effect of psychotherapy in patients with PD: a controlled study. *Parkinsonism Relat. Disord*. **16,** 298–300.
10. Earhart, G.M. (2009) Dance as therapy for individuals with Parkinson disease. *Eur. J. Phys. Rehabil. Med.* **45,** 231–238.
11. Pacchetti, C., Mancini, F., Aglieri, R., Fundaro, C., Martignoni, E., and Nappi, G. (2000) Active music therapy in Parkinson's disease: an integrative method for motor and emotional rehabilitation. *Psychosom. Med*. **62,** 386–393.
12. Reed, K.L. and Sanderson, S.N. (1992) *Concepts of Occupational Therapy*. Williams and Wilkins, Baltimore, MD.
13. Dixon, L., Duncan, D., Johnson, P., Kirkby, L., O'Connell, H., Taylor, H., and Deane, K.H. (2007) Occupational therapy for patients with Parkinson's disease. *Cochrane Database Syst. Rev.* **(3),** CD002813.
14. Mazzoni, P., Hristova, A., and Krakauer, J.W. (2007) Why don't we move faster? Parkinson's disease, movement vigor, and implicit motivation. *J. Neurosci*. **27,** 7105–7116.
15. Forsaa, E.B., Larsen, J.P., Wentzel-Larsen, T., Herlofson, K., and Alves, G. (2008) Predictors and course of health-related quality of life in Parkinson's disease. *Mov. Disord*. **23,** 1420–1427.
16. Bonvin, C., Horvath, J., Christe, B., Landis, T., and Burkhard, P.R. (2007) Compulsive singing: another aspect of punding in Parkinson's disease. *Ann. Neurol*. **62,** 525–528.
17. Evans, A.H., Strafella, A.P., Weintraub, D., and Stacy, M. (2009) Impulsive and compulsive behaviors in Parkinson's disease. *Mov. Disord*. **24,** 1561–1570.
18. Antonini, A. and Cilia, R. (2009) Behavioural adverse effects of dopaminergic treatments in Parkinson's disease: incidence, neurobiological basis, management and prevention. *Drug Saf.* **32,** 475–488.
19. Lemke, M.R., Brecht, H.M., Koester, J., Kraus, P.H., and Reichmann, H. (2005) Anhedonia, depression, and motor functioning in Parkinson's disease during treatment with pramipexole. *J. Neuropsychiatry Clin. Neurosci*. **17,** 214–220.
20. Herman, T., Giladi, N., Gruendlinger, L., and Hausdorff, J.M. (2007) Six weeks of intensive treadmill training improves gait and quality of life in patients with Parkinson's disease: a pilot study. *Arch. Phys. Med. Rehabil*. **88,** 1154–1158.
